# Publisher Correction: Expanding the binding specificity for RNA recognition by a PUF domain

**DOI:** 10.1038/s41467-022-31463-5

**Published:** 2022-06-27

**Authors:** Wei Zhou, Daniel Melamed, Gabor Banyai, Cindy Meyer, Thomas Tuschl, Marvin Wickens, Junyue Cao, Stanley Fields

**Affiliations:** 1 Department of Genome Sciences, University of Washington, Seattle, Washington, USA; 2 Molecular and Cellular Biology Program, University of Washington, Seattle, Washington, USA; 3 The Rockefeller University, New York, NY USA; 4 Department of Evolutionary and Environmental Biology, University of Haifa, Haifa, Israel; 5 Institute of Evolution, University of Haifa, Haifa, Israel; 6 Department of Biochemistry, University of Wisconsin-Madison, Madison, WI USA; 7 Department of Medicine, University of Washington, Seattle, Washington, USA

**Keywords:** Biological techniques, Biotechnology

Correction to: *Nature Communications* 10.1038/s41467-021-25433-6, published online 24 August 2021.

The original HTML version of this Article was updated after publication because the previous HTML version was linked to an incorrect Transparent Peer Review file.

## Supplementary information

Peer Review File

