# Supplementary material for: Expanding the binding specificity for RNA recognition by a PUF domain
Source: Nat Commun. 2021 Aug 24;12:5107. doi: 10.1038/s41467-021-25433-6 (PMC8384837; doi:10.1038/s41467-021-25433-6)
Supplement: Supplementary file 1 — supplementary info [file 41467_2021_25433_MOESM1_ESM.pdf]

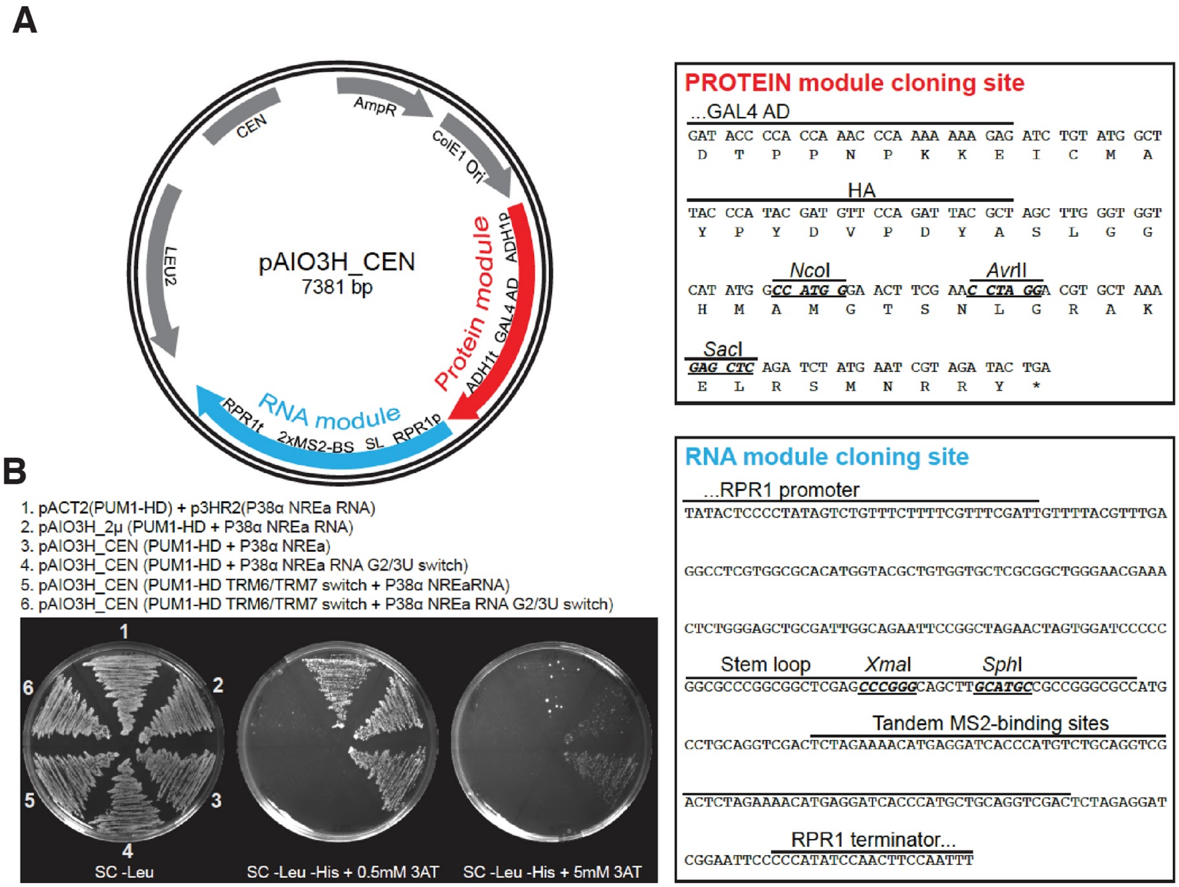

Supplementary Figure 1. (A) An “all-in-one” plasmid that includes both a protein module encoding the human Pumilio-1 PUF domain fused to the Gal4 activation domain (PUF-AD) and an RNA module encoding the Pumilio-1 RNA recognition sequence fused to the MS2 coat protein binding sequence. The cloning sites for both protein module and RNA module are shown on the right. (B) A proof of principle three-hybrid selection experiment in yeast strain YBZ-1. Yeast cells carrying each combination of RNA and the PUF domain grew equally well on media that was used only for selection of the plasmid marker (SC-Leu). In contrast, on media that selects for *HIS3* expression (SC-Leu-His + 0.5mM 3-AT; SC-Leu-His + 5mM 3-AT), the only cells that grew contained a combination of the PUF domain with its wild type RNA target.

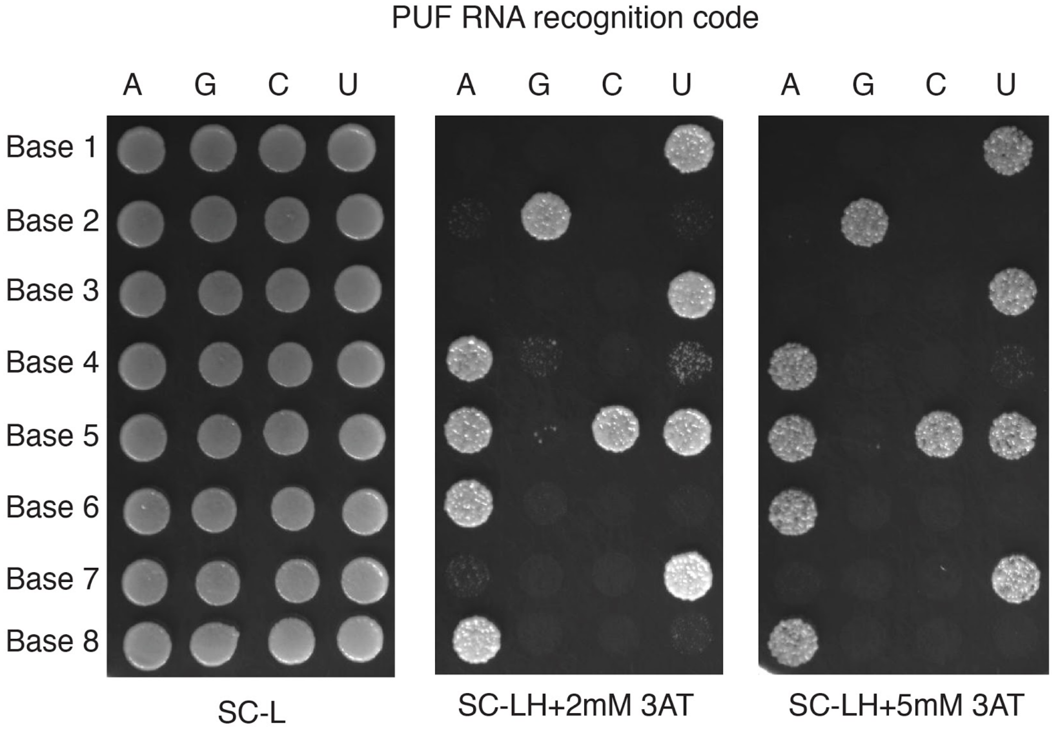

Supplementary Figure 2. The base-specific binding pattern for each wild type PUF repeat is tested through a yeast three-hybrid assay with the “all-in-one” plasmid vector. The left panel (SC-L) shows growth on rich media and the right two panels are selection media (SC-LH+ 2mM 3-AT and SC-L-H + 5mM 3AT). “Base” indicates the location in the recognition RNA sequence; the wild type RNA sequence is 5’-UGUAAAUA-3’.

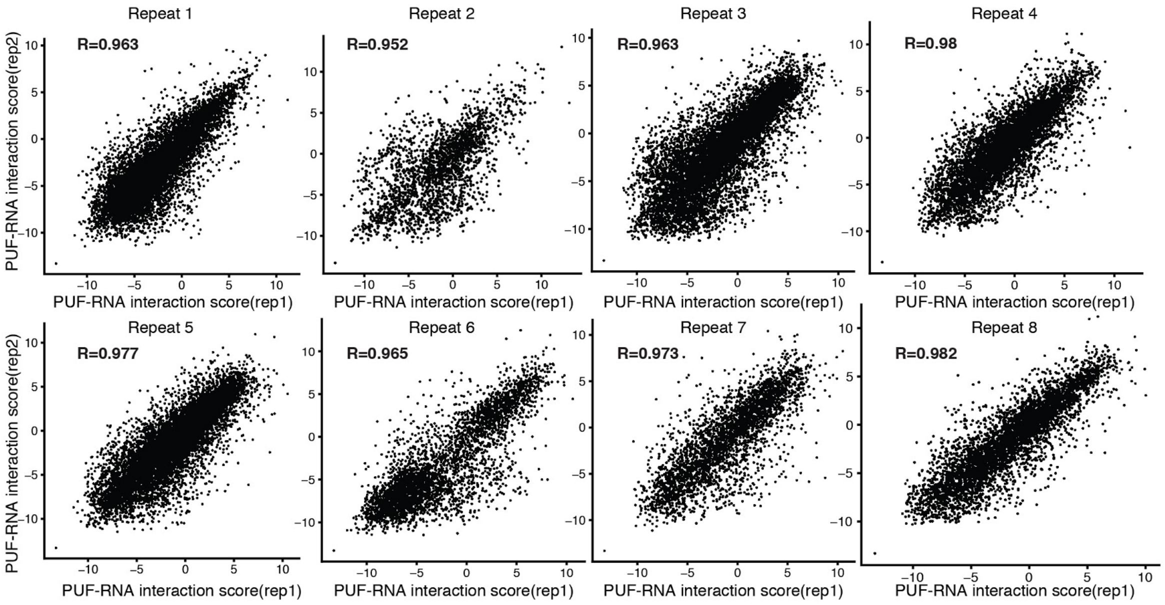

Supplementary Figure 3. Correlation analysis of RNA interaction score for each PUF variant across two experimental replicates from repeat 1 to 8. R indicates the Pearson correlation coefficient.

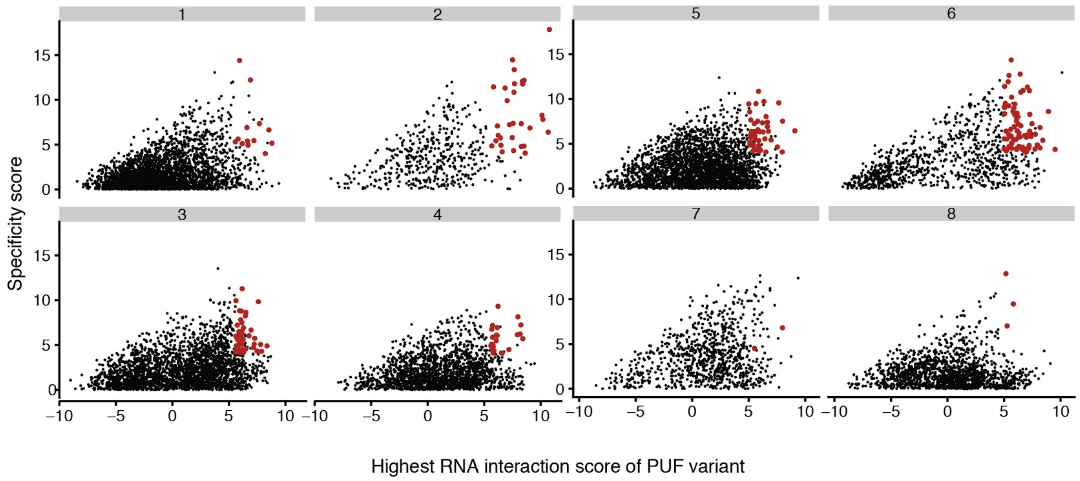

Supplementary Figure 4. Plots showing the RNA interaction score and specificity score of each PUF variant from repeat 1 to repeat 8. The X-axis indicates the highest RNA interaction score of each PUF variant. The Y-axis indicates the specificity score for each PUF variant. Each dot indicates one PUF variant and the red dots highlight those PUF variants with interaction score >5 and specificity score >4.

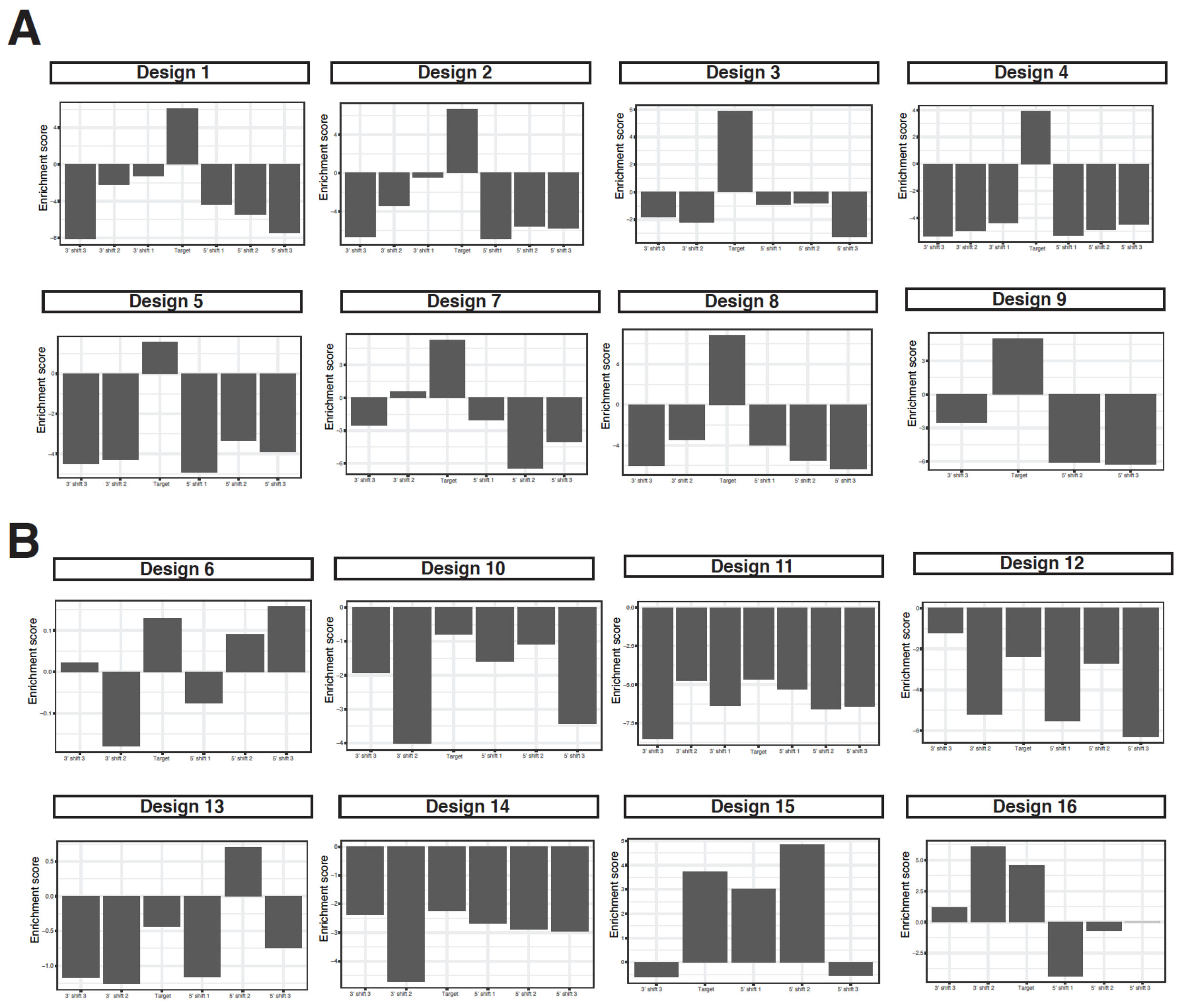

Supplementary Figure 5. Plots showing the enrichment score of different 8-mers for all 16 designs. Y-axis indicates the enrichment score. X-axis indicates the target RNA sequence for the design and the 8-mer RNA sequences upon 1, 2 or 3 base shifts in either the 5’ or 3’direction.
