# Supplementary material for: Expanding the binding specificity for RNA recognition by a PUF domain
Source: Nat Commun. 2021 Aug 24;12:5107. doi: 10.1038/s41467-021-25433-6 (PMC8384837; doi:10.1038/s41467-021-25433-6)
Supplement: Supplementary file 6 — Description of additional supplementary files [file 41467_2021_25433_MOESM6_ESM.docx]

Description of additional supplementary files

Title: Supplementary table 1

Description: Candidate PUF variants chosen for targeted oligonucleotide synthesis

Title: Supplementary table 2

Description: Oligo sequences for targeted RNA element synthesis

Title: Supplementary table 3

Description: Primer sequences used for PUF library preparation
