# Supplementary material for: Expanding the binding specificity for RNA recognition by a PUF domain
Source: Nat Commun. 2021 Aug 24;12:5107. doi: 10.1038/s41467-021-25433-6 (PMC8384837; doi:10.1038/s41467-021-25433-6)
Supplement: Supplementary file 7 — Reporting Summary [file 41467_2021_25433_MOESM7_ESM.pdf]

Corresponding author(s): Stanley Fields

Last updated by author(s): Jul 30, 2021

Reporting Summary

Nature Portfolio wishes to improve the reproducibility of the work that we publish. This form provides structure for consistency and transparency in reporting. For further information on Nature Portfolio policies, see our Editorial Policies and the Editorial Policy Checklist.

Statistics

For all statistical analyses, confirm that the following items are present in the figure legend, table legend, main text, or Methods section.

n/a	Confirmed	
☐	☒	The exact sample size (*n*) for each experimental group/condition, given as a discrete number and unit of measurement
☐	☒	A statement on whether measurements were taken from distinct samples or whether the same sample was measured repeatedly
☒	☐	The statistical test(s) used AND whether they are one- or two-sided<br>*Only common tests should be described solely by name; describe more complex techniques in the Methods section.*
☐	☒	A description of all covariates tested
☒	☐	A description of any assumptions or corrections, such as tests of normality and adjustment for multiple comparisons
☐	☒	A full description of the statistical parameters including central tendency (e.g. means) or other basic estimates (e.g. regression coefficient) AND variation (e.g. standard deviation) or associated estimates of uncertainty (e.g. confidence intervals)
☒	☐	For null hypothesis testing, the test statistic (e.g. *F*, *t*, *r*) with confidence intervals, effect sizes, degrees of freedom and *P* value noted<br>*Give P values as exact values whenever suitable.*
☒	☐	For Bayesian analysis, information on the choice of priors and Markov chain Monte Carlo settings
☒	☐	For hierarchical and complex designs, identification of the appropriate level for tests and full reporting of outcomes
☐	☒	Estimates of effect sizes (e.g. Cohen's *d*, Pearson's *r*), indicating how they were calculated

Our web collection on statistics for biologists contains articles on many of the points above.

Software and code

Policy information about availability of computer code

Data collection	Base calls were converted to fastq format and demultiplexed using Illumina's bcl2fastq/ 2.16.0.10 tolerating one mismatched base in barcodes (edit distance (ED) < 2). Read1 and read2 are merged using SeqPrep/2016. Low quality reads were filtered based on the exact match of the first 10bp common sequence in the plasmid.
Data analysis	We determined the frequency of each variant in both input pool and post-selection pool by comparing its sequencing reads from both pools. The log2 change in the frequency from input to selection pool serves as a measure of binding activity for each PUF variant, designated as a PUF domain–RNA interaction score in this assay.<br><br>For the in vitro binding assay analysis, the Kd value was determined by first using ImageJ software to quantify the signal in each RNA band. Background signals from blank regions of the gel were subtracted from the signal intensities obtained from the bands. The fraction of RNA bound was determined from the background-subtracted signal intensities using the expression: bound/(bound + unbound). The fraction of RNA bound in each reaction was plotted versus the concentration of PUF protein. We then used Prism Software to perform non-linear regression and obtain a value for Kd and its 95% CI.

For manuscripts utilizing custom algorithms or software that are central to the research but not yet described in published literature, software must be made available to editors and reviewers. We strongly encourage code deposition in a community repository (e.g. GitHub). See the Nature Portfolio guidelines for submitting code & software for further information.

Data

Policy information about availability of data

All manuscripts must include a data availability statement. This statement should provide the following information, where applicable:

- Accession codes, unique identifiers, or web links for publicly available datasets
- A description of any restrictions on data availability
- For clinical datasets or third party data, please ensure that the statement adheres to our policy

High-throughput sequencing reads, along with datasets showing calculated interaction scores for both random screening and targeted screening, have been submitted to Gene Expression Omnibus (GEO) under accession nos: GSE152452 and they are currently available to the public. In addition, the crystal structure of the Pumilio-homology domain and RNA interaction is available online in database PDB ID 1M8Y.

Field-specific reporting

Please select the one below that is the best fit for your research. If you are not sure, read the appropriate sections before making your selection.

☒ Life sciences ☐ Behavioural & social sciences ☐ Ecological, evolutionary & environmental sciences

For a reference copy of the document with all sections, see nature.com/documents/nr-reporting-summary-flat.pdf

Life sciences study design

All studies must disclose on these points even when the disclosure is negative.

Sample size	For each selection, we have 2000-5000 colonies and ~10 million reads for each input and selected sample. We choose this sample size as these colony size can sample the majority of PUF-RNA interactions and the sequencing reads are sufficient to recover all major interactions
Data exclusions	no data were excluded from the study.
Replication	We performed 2 replicates for the first experiment, 1 replicate for the second experiment(which aims to valid the first experiment result), 2 replicates for the third experiment. All attempts at replication are successful and replicates from each experiment highly correlate wiht each other.
Randomization	This is not relevant to our study - this is a selection experiment started with a randomized DNA library.
Blinding	No blinding is included - this is a selection experiment that we start from a randomized DNA library and Y3H selection media is used to select for the interaction.

Reporting for specific materials, systems and methods

We require information from authors about some types of materials, experimental systems and methods used in many studies. Here, indicate whether each material, system or method listed is relevant to your study. If you are not sure if a list item applies to your research, read the appropriate section before selecting a response.

Materials & experimental systems

n/a	Involved in the study	
☒	☐	Antibodies
☐	☒	Eukaryotic cell lines
☒	☐	Palaeontology and archaeology
☒	☐	Animals and other organisms
☒	☐	Human research participants
☒	☐	Clinical data
☒	☐	Dual use research of concern

Methods

n/a	Involved in the study	
☒	☐	ChIP-seq
☒	☐	Flow cytometry
☒	☐	MRI-based neuroimaging

Eukaryotic cell lines

Policy information about cell lines

Cell line source(s)	Yeast YBZ strain was used
Authentication	The key marker genes and reporter genes were authenticated

Mycoplasma contamination	No contamination
Commonly misidentified lines (See ICLAC register)	does not apply to this study.
