# Supplementary figures and images for: Expanding the binding specificity for RNA recognition by a PUF domain
Source: Nat Commun. 2021 Aug 24;12:5107. doi: 10.1038/s41467-021-25433-6 (PMC8384837; doi:10.1038/s41467-021-25433-6)

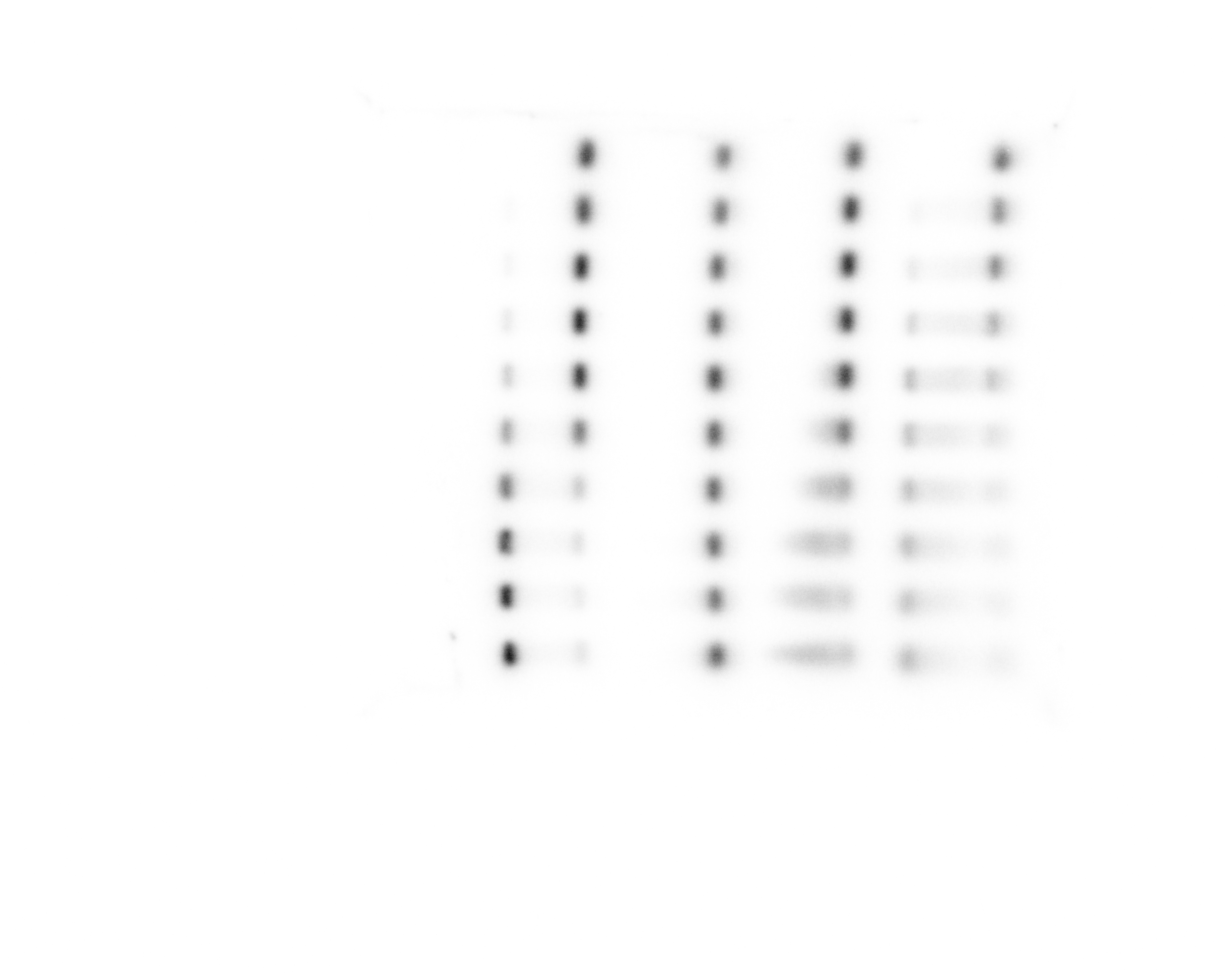

Supplement: Supplementary file 8 — Source data file [file 41467_2021_25433_MOESM8_ESM.zip › source Data/20210420_Pum_EMSA_wt_34_ON_eposure_Repl1-[Phosphor].tif]

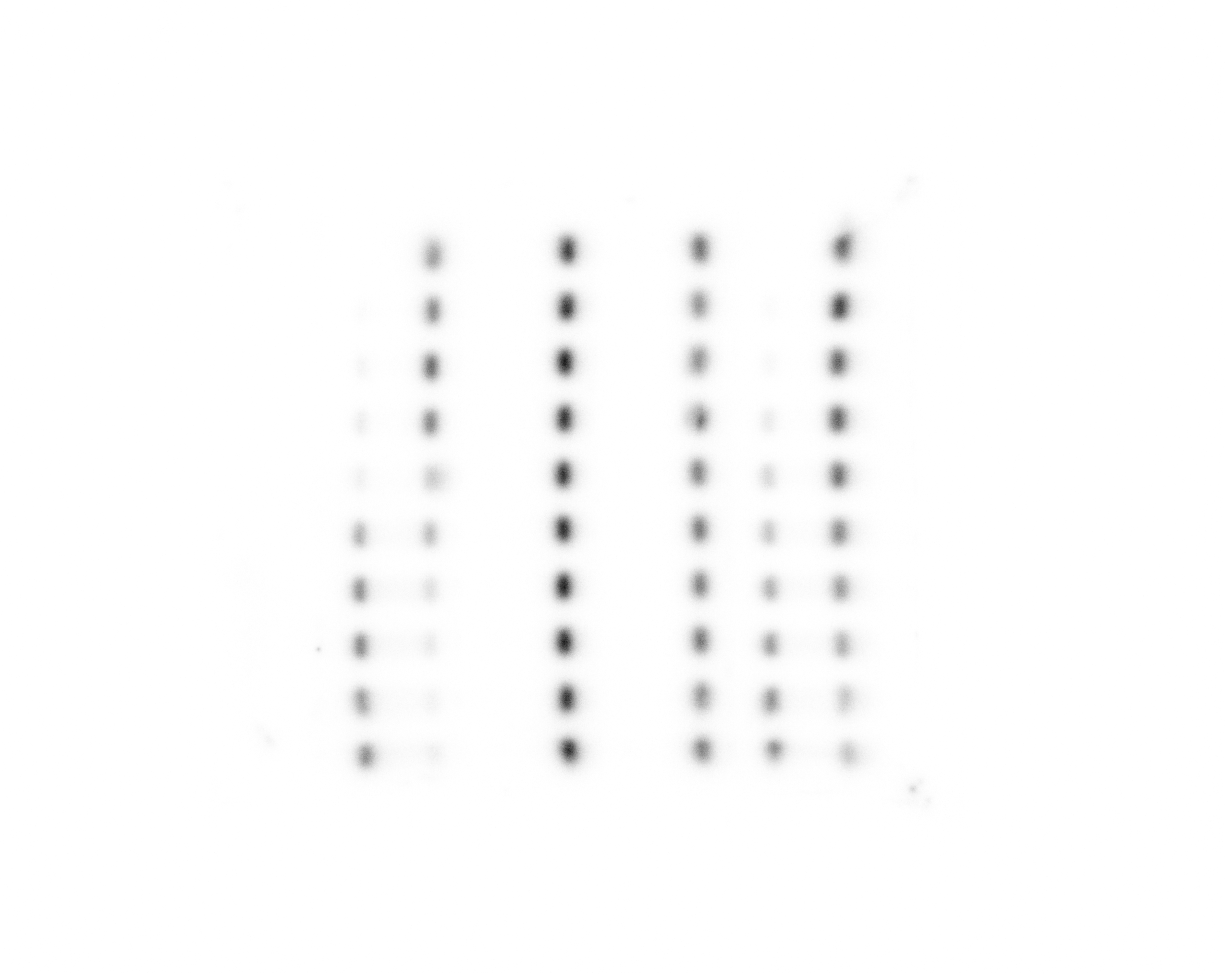

Supplement: Supplementary file 8 — Source data file [file 41467_2021_25433_MOESM8_ESM.zip › source Data/20210421_Pum_EMSA_wt_12_ON_eposure_Repl2-[Phosphor].tif]

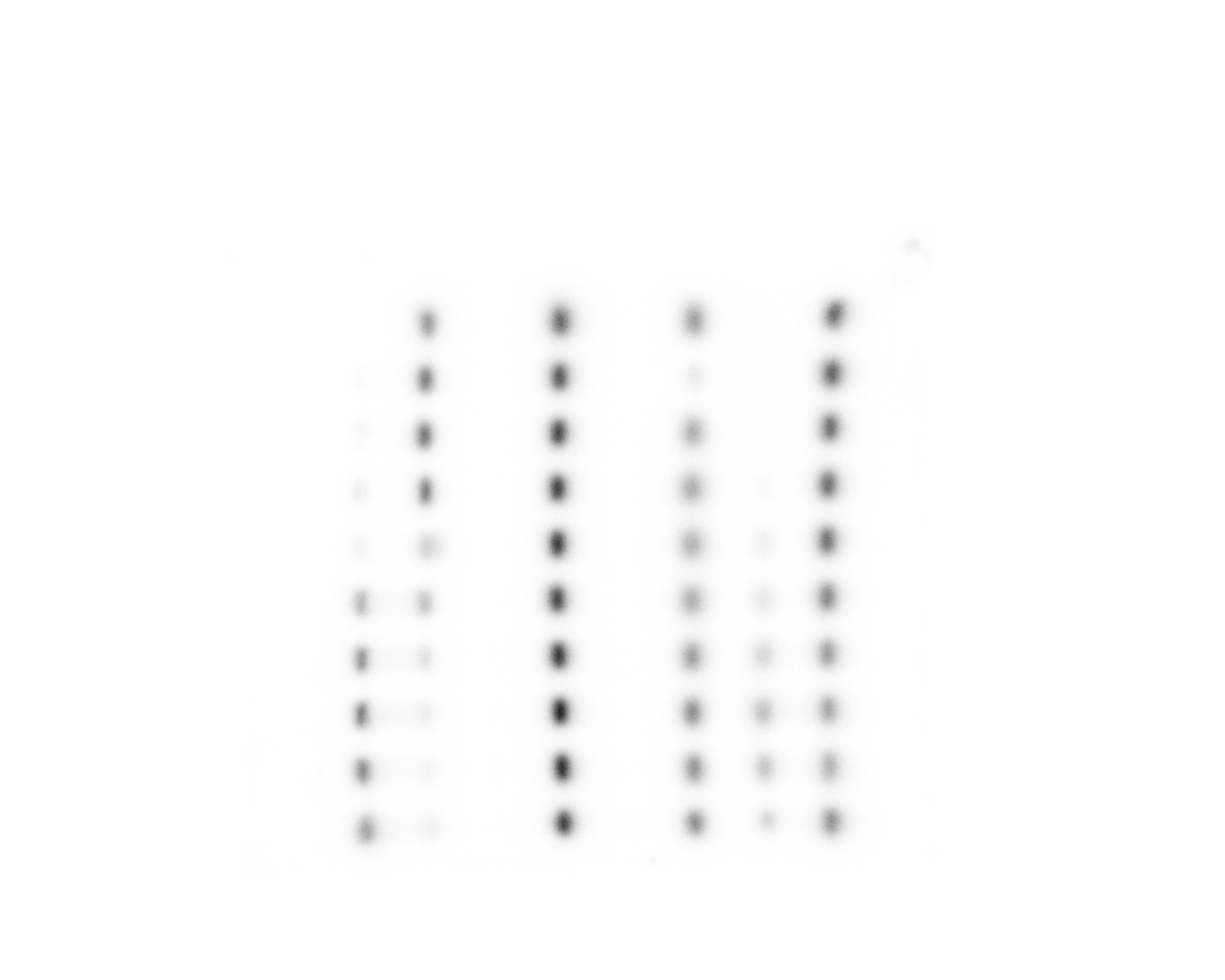

Supplement: Supplementary file 8 — Source data file [file 41467_2021_25433_MOESM8_ESM.zip › source Data/20210421_Pum_EMSA_wt_12_ON_eposure_Repl1-[Phosphor].tif]

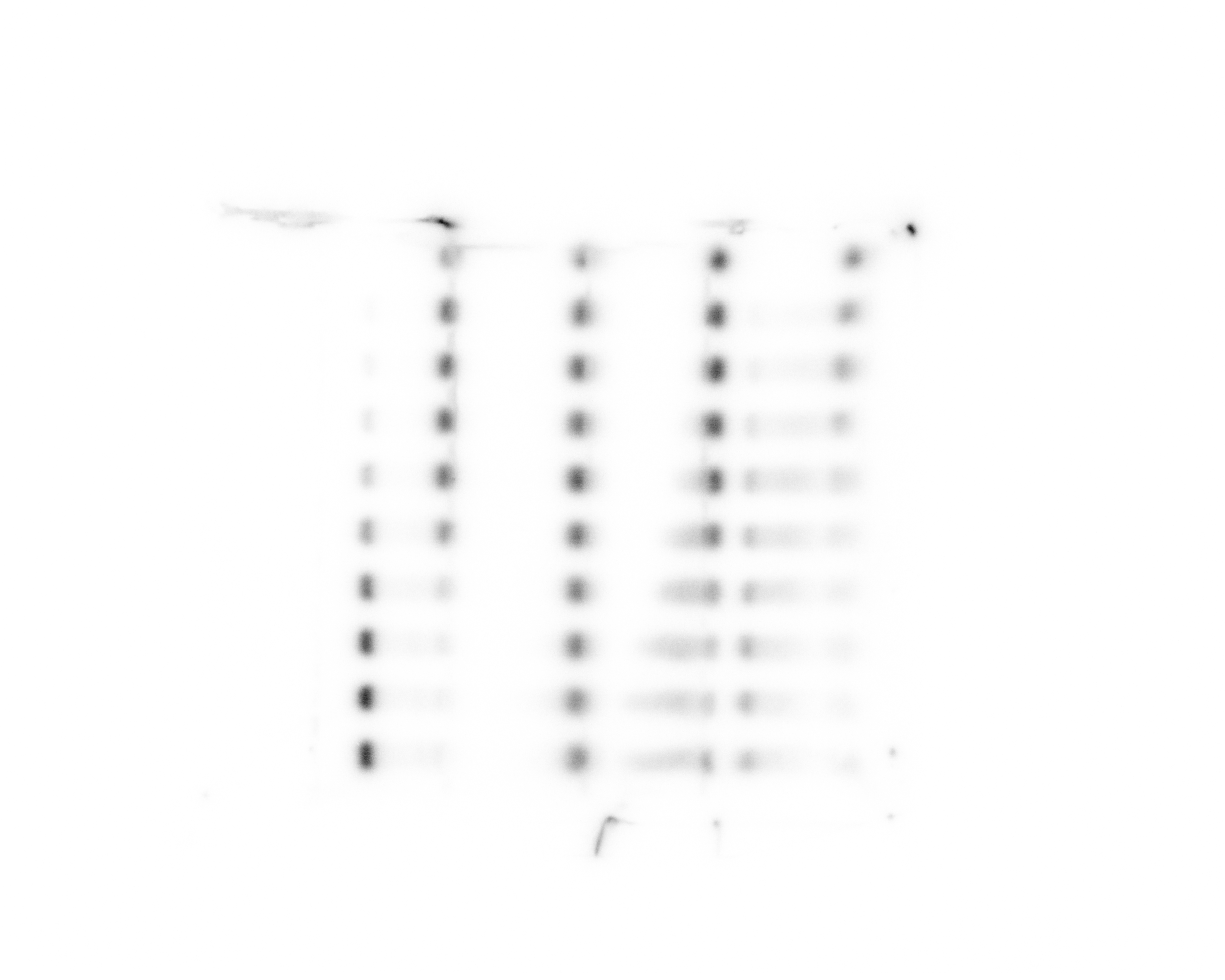

Supplement: Supplementary file 8 — Source data file [file 41467_2021_25433_MOESM8_ESM.zip › source Data/20210420_Pum_EMSA_wt_34_ON_eposure_Repl2-[Phosphor].tif]
